# Differential effects of reticulophagy and mitophagy on nonalcoholic fatty liver disease

**DOI:** 10.1038/s41419-017-0136-y

**Published:** 2018-01-24

**Authors:** Lijun Pang, Kai Liu, Daojie Liu, Fudong Lv, Yunjin Zang, Fang Xie, Jiming Yin, Ying Shi, Yanjun Wang, Dexi Chen

**Affiliations:** 1 Beijing Institute of Hepatology, Capital Medical University, 100069 Beijing, China; 2 Beijing You’an Hospital, Capital Medical University, 100069 Beijing, China; 3 Department of Clinical Laboratory, Haidian Maternal & Child Health Hospital, 100080 Beijing, China; 4 Organ Transplantation Center, The Affiliated Hospital of Qingdao University, 26603 Qingdao, Shandong China

## Abstract

Autophagy affects the pathological progression of non-alcoholic fatty liver disease (NAFLD); however, the precise role of autophagy in NAFLD remains unclear. In this study, we want to identify the role of autophagy including reticulophagy and mitophagy in NAFLD pathogenesis. When HepG2 cells were treated with 400 μM oleic acid (OA), increased reticulophagy was induced 8 h after treatment, which correlated with an anti-apoptotic response as shown by the activation of the PI3K/AKT pathway, an increase in BCL-2 expression, and the downregulation of OA-induced lipotoxicity. When treated with OA for 24 h, DRAM expression-dependent mitophagy resulted in increased apoptosis in HepG2 cells. Inhibition of reticulophagy aggravated and increased lipotoxicity-induced apoptosis 8 h after treatment; however, the inhibition of mitophagy decreased hepatocyte apoptosis after 24 h of OA treatment. Results from the analysis of patient liver samples showed that autophagic flux increased in patients with mild or severe NAFL. PI3K/AKT phosphorylation was observed only in samples from patients with low-grade steatosis, whereas DRAM expression was increased in samples from patients with high-grade steatosis. Together, our results demonstrate that reticulophagy and mitophagy are independent, sequential events that influence NAFLD progression, which opens new avenues for investigating new therapeutics in NAFLD.

## Introduction

Non-alcoholic fatty liver disease (NAFLD) is a very common public health problem that affects approximately one-third of adults and an increasing number of children in developed countries^1^. NAFLD is most likely promoted by the deregulation of hepatic lipid metabolism as a hepatic manifestation of metabolic syndrome, which clusters visceral obesity, dyslipidemia, hypertension, atherosclerosis, and insulin resistance^2^. NAFLD can progress from simple steatosis (i.e., non-alcoholic fatty liver, NAFL) to non-alcoholic steatohepatitis (NASH), liver fibrosis, cirrhosis, and even hepatocellular carcinoma^3^. The exact mechanisms of the NAFL-to-NASH transition remains elusive; however, this transition is a crucial point for NAFL due to the increase in morbidity and mortality upon the development of NASH^4^. The most prevalent mechanism used to explain the development of NASH is the “multiple hit” hypothesis^5^. According to the multiple hit hypothesis, various hepatic insults result in lipid deregulation and steatosis, as the accumulation of lipid is sufficient to induce lipid peroxidation and inflammation associated with the NAFL-to-NASH transition^6^.

Autophagy-dependent degradation of compromised organelles is a cellular pathway crucial for maintaining cell homeostasis, and organellar autophagy can be selective for the degradation of mitochondria (mitophagy), endoplasmic reticulum (reticulophagy), and lipid droplets (lipophagy)^7–9^. Lipophagy is currently regarded as an alternative pathway for lipid metabolism in hepatocytes, and the impairment of lipophagy contributes to NAFLD development^7^. Previous studies have shown that free fatty acid (FFA)-induced lipotoxicity impairs the function of the endoplasmic reticulum (ER) by increasing protein misfolding and by inducing ER stress, which can exacerbate NAFLD^10^. Furthermore, autophagy can lead to cell death and has been shown to be associated with caspase-3 activation-dependent apoptosis^10,11^. However, the exact mechanisms by which mitophagy, reticulophagy, and lipophagy influence NAFLD progression remain unknown.

Activation of the PI3K/AKT signaling pathway is involved in protecting cells from apoptosis, whereas the activation of the p53 signaling pathway promotes apoptosis^12^. However, both pathways affect the activation of autophagy and have been reported to be associated with NAFLD development^13,14^. PI3K/AKT pathway activation is thought to regulate autophagy through mTOR, and the PI3K inhibitor LY294002 is a general inhibitor for autophagic sequestration^13,15^. However, class III PI3K (VPS34) appears to be involved in autophagosome biogenesis^13^. Following genotoxic stress, p53 becomes activated and inhibits cell proliferation in an effort to maintain genomic integrity^16^. p53 functions primarily as a transcription factor and induces the expression of several p53 target genes such as p21 and PUMA (p53 up-regulated modulator of apoptosis). p21 inhibits several cyclin-dependent kinases, which results in either G1 arrest or G2/M checkpoint arrest. PUMA is a pro-apoptotic BH3-only BCL-2 family protein that promotes BCL2-associated X protein (BAX) expression and mitochondria-dependent apoptosis^17^. A relationship between the p53 signaling pathway and autophagy activation was established due in part to the discovery of the p53-dependent regulation of DRAM (damage-regulated autophagy modulator) and Sestrin2, which were subsequently shown to modulate autophagy and promote apoptosis^18–20^. However, basal levels of cytoplasm-resident p53 also appear to directly inhibit autophagy^21^.

Considering that lipophagy can regulate lipid metabolism, we hypothesized that autophagy might also play a role in promoting resistance to damage caused by high levels of FFA. However, we failed to identify lipophagy as a contributing factor in the reduction of lipid droplet formation in HepG2 cells subjected to 400 μM oleic acid (OA) treatment. In contrast, OA treatment primarily induced reticulophagy, which prevented hepatocytes from undergoing lipotoxicity-induced apoptosis apparently through a decrease in lipotoxicity, the activation of the PI3K/AKT pathway, and an increase in BCL-2 expression. After reticulophagy subsided, OA treatment induced mitophagy, which was mediated by DRAM and played a pro-apoptotic role resulting in hepatocyte death. Furthermore, we showed that autophagy could be identified in liver samples from patients with low- and high-grade steatosis. However, PI3K/AKT activation could only be identified in liver tissues from patients with low-grade steatosis, whereas DRAM expression could only be identified in liver tissues from patients with high-grade steatosis. The results of our clinical specimen analysis support our in vitro data, which suggest that reticulophagy and mitophagy may be independent, sequential events responsible for the pathological progression of NAFLD, which can trigger both anti- and pro-apoptotic outcomes in hepatocytes.

## Results

### Two waves of autophagy can be induced in HepG2 cells at different stages of NAFLD in response to OA stimulus

**Fig. 1:**
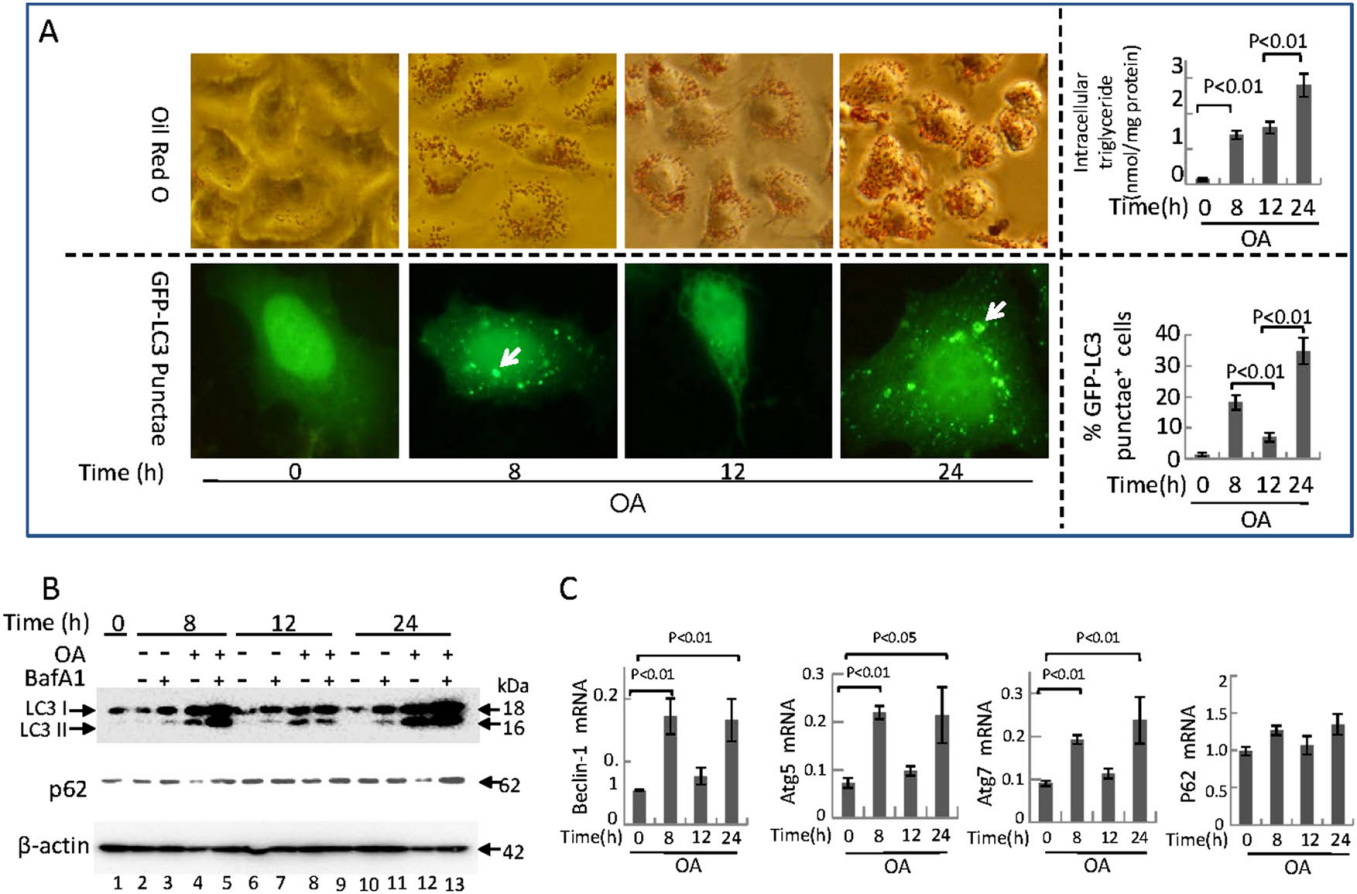
Autophagy development peaks at 8 and 24 h in response to 400 μM OA stimulus in HepG2 cells **a** Oil Red O staining (upper left panel, original magnification, ×400) and intracellular triglyceride levels of cells (upper right panel); representative GFP-LC3 images (lower left panel, original magnification, ×1000) and the percentage of autophagosomes (lower right panel) of the cells. White arrow indicates autophagosomes formation and cells with 5 or more GFP-LC3 puncta were considered to have accumulated autophagosomes. Data are presented as mean ± SEM in three independent experiments. **b** Representative western blotting analysis of LC3I/II and p62 expressions after using Bafilomycin A1 in cells. **c** Real-time PCR analysis of mRNA levels of Beclin-1, Atg5 a, Atg7, and p62 in cells. Data are presented as mean ± SEM in three independent experiments

**Fig. 2:**
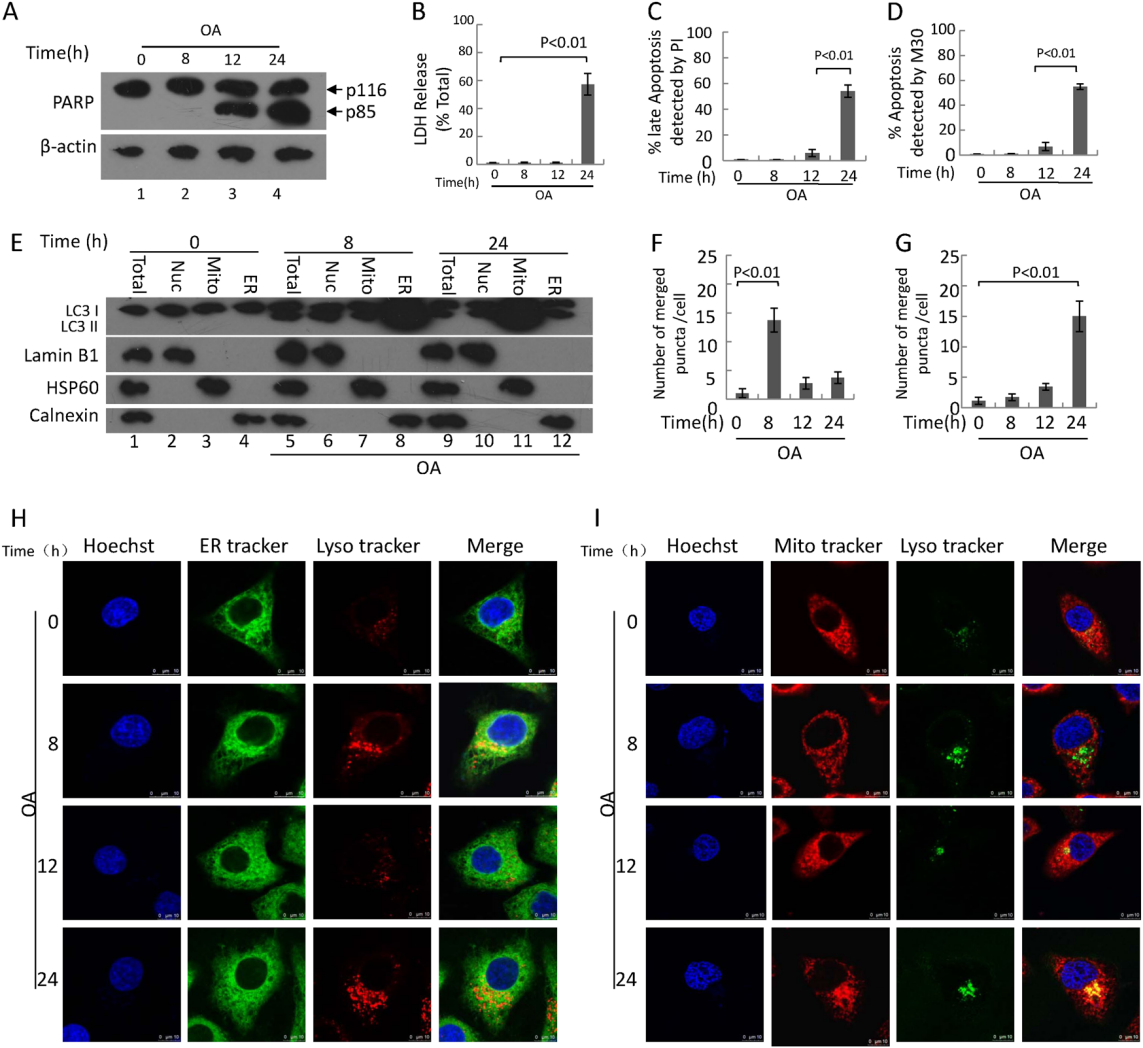
OA treatment induces apoptosis, cell impairment, reticulophagy, and mitophagy development HepG2 cells were treated with 400 μM OA for 24 h. **a** Representative western blotting analysis of cleaved PARP fragment in cells. **b** The levels of LDH release in supernatant. **c**, **d** Quantification of apoptotic cells by Calcein AM/PI(B) and M30(C) immunoreactivity. Data are presented as mean ± SEM in three independent experiments. **e** Subcellular fractions of HepG2 cells were subjected to a western blotting assay with indicated antibodies. Lamin B1, HSP60, and calnexin were used as controls for the nuclei (Nuc), mitochondrial (Mito), and endoplasmic reticulum (ER) fractions, respectively. Total: total lysate of cells. **h** The degree of colocalization of endoplasmic reticulum with lysosomes in HepG2 cells was measured via live-cell imaging microscopy. LysoTracker Red DND-99 staining was applied to mark lysosomal structures (red), and ER-Tracker Green to visualize endoplasmic reticulum (green). Hoechst 33342 dye was used to stain nuclei (blue). A positive colocalization is indicated by yellow signals (merge) due to the overlap of LysoTracker Red and ER-Tracker Green staining. Scale bars: 10 μm. **i** The degree of colocalization of mitochondria with lysosomes in HepG2 cells. LysoTracker was green pseudo, and MitoTracker was red. A positive colocalization is indicated by yellow signals (merge) due to the overlap of LysoTracker Red and MitoTracker Green staining. Scale bars: 10 μm. **f**, **g** Quantification of the merged number which was yellow. Data are presented as mean ± SEM in three independent experiments

Autophagy plays a pivotal role in regulating the pathological progression of NAFLD^22^; however, the mechanism remains incompletely understood. Therefore, we sought to identify the role of FFA-induced autophagy on NAFLD development using an in vitro model. Previous studies have shown that OA induces NASH development in HepG2 cells, which is characterized by the accumulation of lipid droplets and the induction of apoptosis at concentrations ranging from 0.1 to 2 mM^23–25^. We treated HepG2 cells with 400 μM OA for 24 h to observe the progression of NAFLD in vitro. The induction of lipid droplet formation was confirmed by Oil Red O staining and by measuring the concentration of intracellular triglycerides (Fig. 1a, b). Moreover, OA significantly increased cell death as determined by the fact that LDH (Lactate Dehydrogenase) release was observed 24 h post-treatment (Fig. 2b). These results suggest that 400 μM OA induces the progression of NAFLD during the 24-h treatment period.

We examined whether autophagy might be associated with the development of NAFLD in HepG2 cells treated with OA. We observed that autophagosome formation reached a peak at 8 and 24 h post-OA treatment (Fig. 1a), and transfection of HepG2 cells with an empty vector showed no significant change (data not shown). Western blotting results also showed LC3-II level was mainly induced upon 8 and 24 h of OA treatment (Fig. 1b). The levels of p62 are contrary to LC3-II, which appear to correlate well with other parameters of autophagic flux^26^. In addition, we examined autophagic flux by using BafilomycinA1 (BafA1), which is used to prevent the fusion of autophagosomes with lysosomes. The two waves of autophagy flux OA-induced were blocked by BafA1 obviously. We also obtained similar results in other two types of hepatocytes including 7702 and SMMC-7721 cells (Supplementary Figure 1). Our results showed that the mRNA levels of beclin-1, Atg5, and Atg7 had two peaks except p62 (Fig. 1d). These results suggest that two waves of autophagy are present in the development of NAFLD. Therefore, we sought to identify the role and mechanism of these two waves of autophagic flux.

### The first wave of autophagy is mainly reticulophagy and plays a role in protecting hepatocytes from apoptosis; the second wave of autophagy is mainly mitophagy and contributes to hepatocytic apoptosis

Because apoptosis contributes to the pathological progression of NAFLD, we investigated possible connections between autophagy and apoptosis in OA-treated HepG2 cells. We found that OA treatment induced the cleavage of PARP (poly ADP-ribose polymerase) upon 12 and 24 h of treatment (Fig. 2a). PARP is cleaved by caspase-3 and caspase-7 to yield p85 and p25 PARP fragments, respectively, and the cleavage of PARP has been widely used as an apoptotic marker^27^. Furthermore, we employed calcein AM/PI and M30 immunoreactivity to assess apoptosis. Calcein AM stains for viable cells, whereas PI stains for late apoptotic cells. M30 stains caspase-3-cleaved keratin 18 and thus identifies apoptotic cells. Recently, increased levels of M30 have also been considered to be a marker of NAFLD development^28–30^. However, HepG2 cell apoptosis was observed 12 h and reached a peak 24 h post-OA treatment (Fig. 2c, d and Supplementary Figure 2).

Stress can induce both reticulophagy and mitophagy in cells^9,31^. The ER senses the accumulation of misfolded proteins caused by lipotoxicity and can induce reticulophagy^31^. Although mitophagy maintains intracellular mitochondrial homeostasis^32,33^, excessive mitochondrial mitophagy may promote cell death^34^. Our results showed the LC3-II/LC3-I ratio in ER-enriched fragments was about 4/1 at 8 h, but the ratio in Mito-enriched fragments was about 4/1 at 24 h (Fig. 2e); there was no significant difference at 12 h (Supplementary Figure 2). PINK1 (PTEN-induced putative kinase1) functions in a common signaling pathway known to regulate mitochondrial network homeostasis and quality control, including mitophagy^35^. The results showed that PINK1 siRNA significantly inhibited autophagy and cell apoptosis at 24 h post-OA treatment (Supplementary Figure 3). According to the guidelines, we detected colocalization of mitochondria or ER with lysosomes^36^. Our results showed that merged dots of ER with lysosomes peaked at 8 h, but the merged dots of mitochondria with lysosomes peaked at 24 h (Fig. 2f–i). These results suggest that the first wave of autophagy occurred mainly in the ER, whereas the second wave occurred mainly in the mitochondria.

### The persistence of the first wave of autophagy downregulates lipotoxicity and hepatocyte death induced by OA

**Fig. 3:**
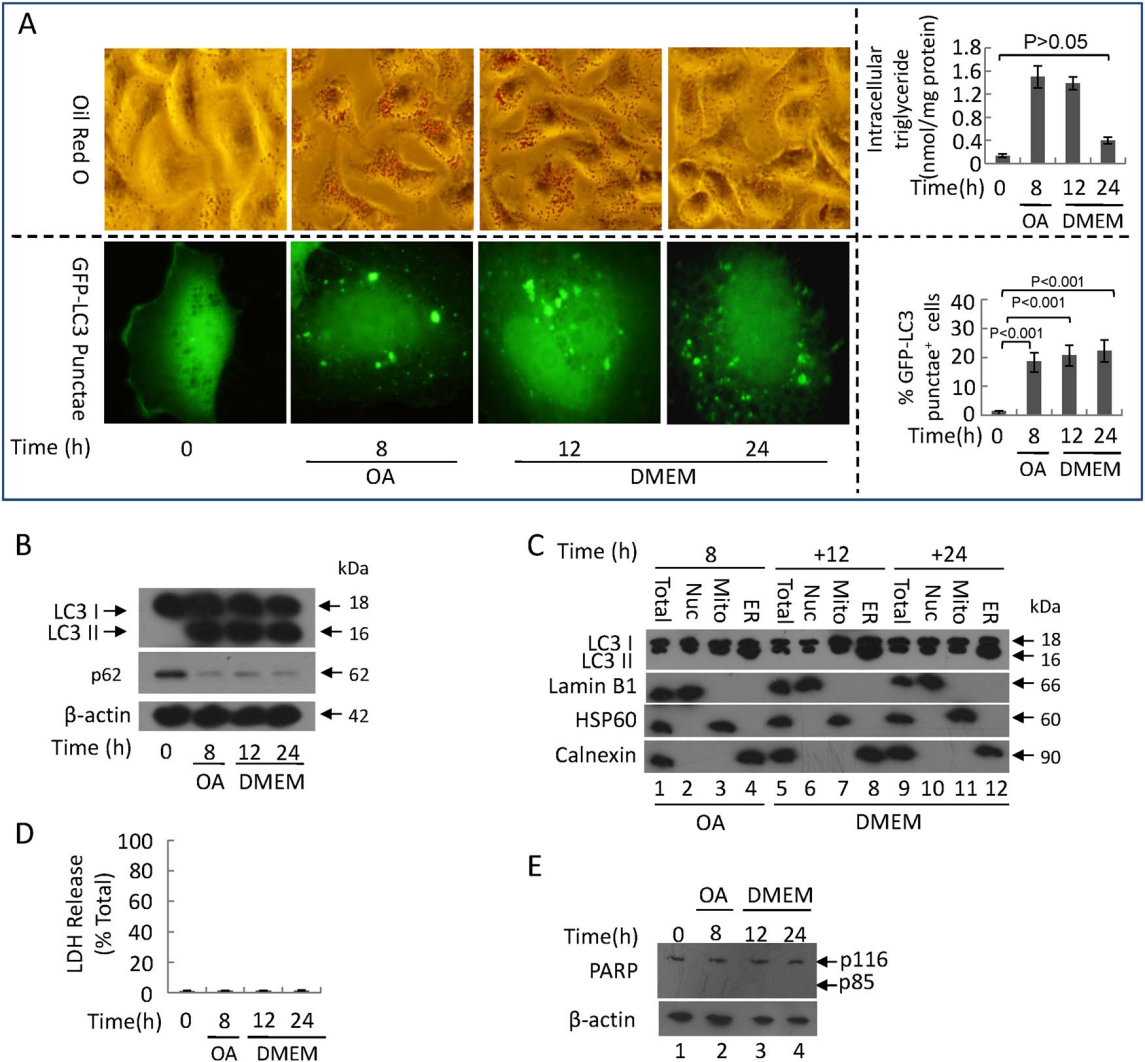
The first wave of autophagy prevents 400 μM OA-induced lipotoxicity and hepatocytic apoptosis HepG2 cells were stimulated by 400 μM OA for 8 h and then were cultured in normal DMEM culture medium for 12 and 24 h. **a** Oil Red O staining (upper left panel, original magnification, ×400) and intracellular triglyceride levels of cells (upper right panel); representative GFP-LC3 images (lower left panel, original magnification, ×1000) and the percentage of autophagosomes (lower right panel) of the cells. Cells with 5 or more GFP-LC3 puncta were considered to have accumulated autophagosomes. Data are presented as mean ± SEM in three independent experiments. **b**, **e** Representative western blotting analysis of LC3 I/II and p62(B) and cleaved PARP fragment **e**. **c** Subcellular fractions were subjected to a western blotting assay with indicated antibodies. Lamin B1, HSP60, and calnexin were used as controls for the nuclei (Nuc), mitochondrial (Mito), and endoplasmic reticulum (ER) fractions, respectively. Total: total lysate of cells. **d** The levels of LDH release in HepG2 cells were analyzed. Data are presented as mean ± SEM in three independent experiments

Removing OA from the culture medium after 8 h of OA treatment, we observed that the levels of lipid droplets and intracellular triglycerides after 8 h OA + 24 h DMED returned to the baseline level (Fig. 3a). Moreover, we examined the effects of removing OA on autophagic flux. Following an 8-h pre-treatment with OA, autophagy was observed 12 and 24 h after the removal of OA (Fig. 3a, b), which coincided with a reduction in lipid droplet formation (Fig. 3a). According to western blotting, we found that LC3-II mainly exists in the ER-enriched fragments from 8 to 24 h after OA withdrawal (Fig. 3c), suggesting that the autophagic process initially consists mainly of reticulophagy following the removal of OA. The persistence of reticulophagy did not induce bulk LDH release at 24 h (Fig. 3d) or the development of apoptosis at 12 and 24 h (Fig. 3e). These data suggest that OA removal extends the persistence of the reticulophagy-predominant phase to 24 h. Importantly, OA withdrawal does not increase cell death and leads to a decrease in the level of lipid droplets, which suggests that reticulophagy during early ER stress is not associated with the induction of cell death. Based on this observation, we investigated the role of reticulophagy in protecting hepatocytes from lipotoxicity-induced apoptosis.

### The first wave of reticulophagy upon OA treatment for 8 h might play a protective role associated with autophagy inhibition, but the second wave of mitophagy has opposite effects

**Fig. 4:**
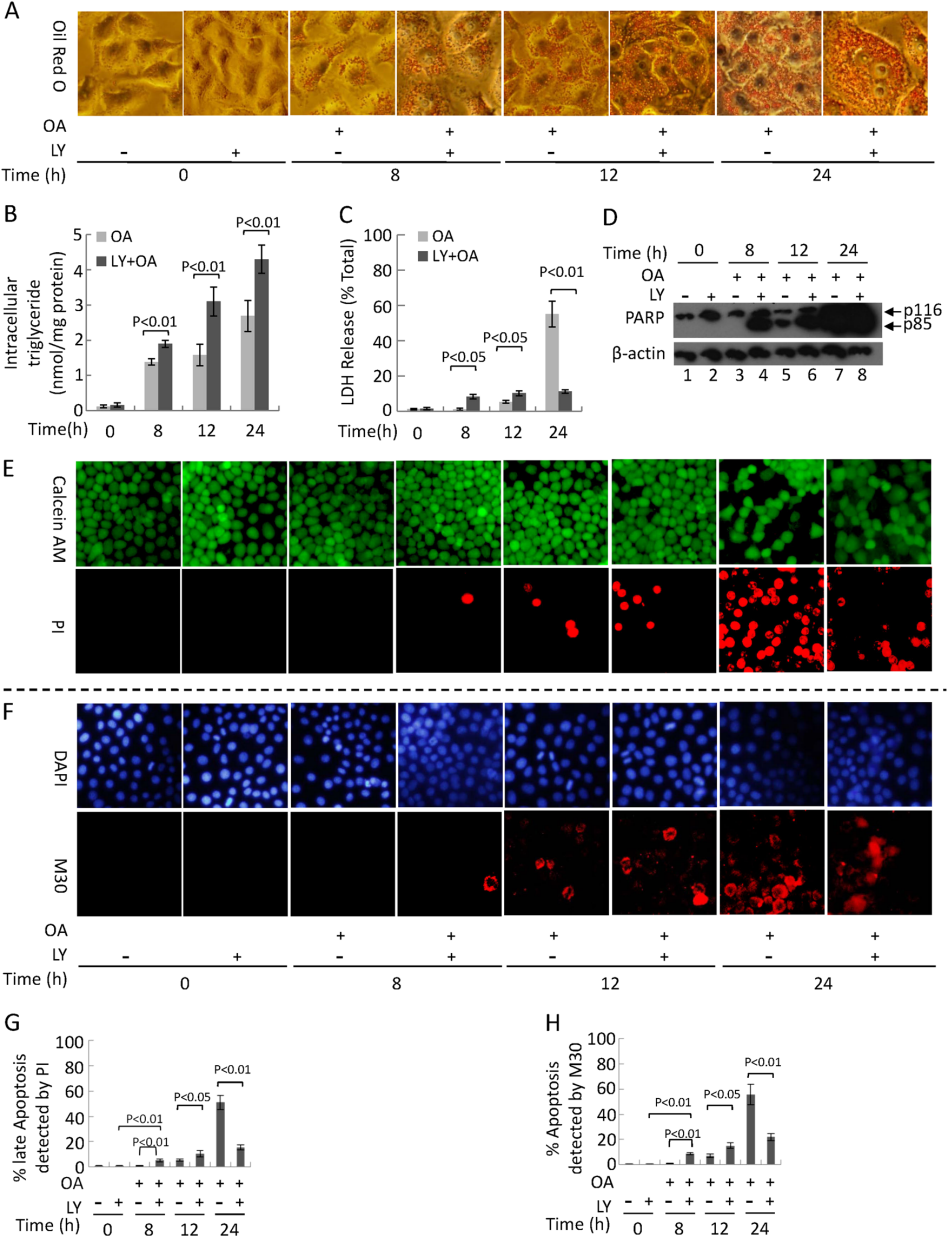
Autophagy inhibition by LY294002 (LY) promotes earlier apoptosis development at 8 h, but decreases the level of apoptosis at 24 h LY294002, a PI3K inhibiter, was added into the culture for 5 h to inhibit autophagy and replaced by 400 μM OA. **a** Oil Red O staining (original magnification, ×400). **b** Intracellular triglyceride levels. **c** The levels of LDH release. **d** Representative western blotting analysis of cleaved PARP fragment in cells. Representative images of calcein AM/PI staining (**e**), DAPI/M30 staining (**f**), and quantification of late (**g**) and early (**h**) apoptosis by PI staining and M30 immunoreactivity, respectively. **b**, **c**,** g**, **h** Data are presented as mean ± SEM in three independent experiments

To elucidate the relationship between the two waves of autophagy and the occurrence of cell death, we used LY294002 to inhibit autophagy. Our results showed that LY294002 pre-treatment induced increasing severe lipotoxicity (Fig. 4a–c). Calcein AM/PI and M30 immunoreactivity assays showed that cell death significantly increased from 8 to 12 h of OA treatment when PI3K was inhibited by LY294002 (Fig. 4e–h). The cleaved PARP fragment (p85) band could be detected upon OA treatment for 8 h (Fig. 4d). These results suggest that autophagy that occurs within the 8-h time frame post-OA treatment might play a protective role. However, the second wave of autophagy inhibited by LY294002 decreased LDH release, and cell death at 24 h of OA treatment suggests that the second wave of autophagy promotes apoptosis (Fig. 4c, e–h). Single agent LY294002 treatment at 50 μM does not induce HepG2 cell death (Supplementary Figure 4). In addition, ATG5 siRNA significantly inhibited autophagy at 8 and 24 h post-OA treatment, and inhibited cell apoptosis distinctly at 24 h (Supplementary Figure 5).

### Activation of the PI3K/AKT signaling pathway is involved in reticulophagy; mitophagy induces HepG2 apoptosis by promoting DRAM expression and by decreasing BCL-2 expression

**Fig. 5:**
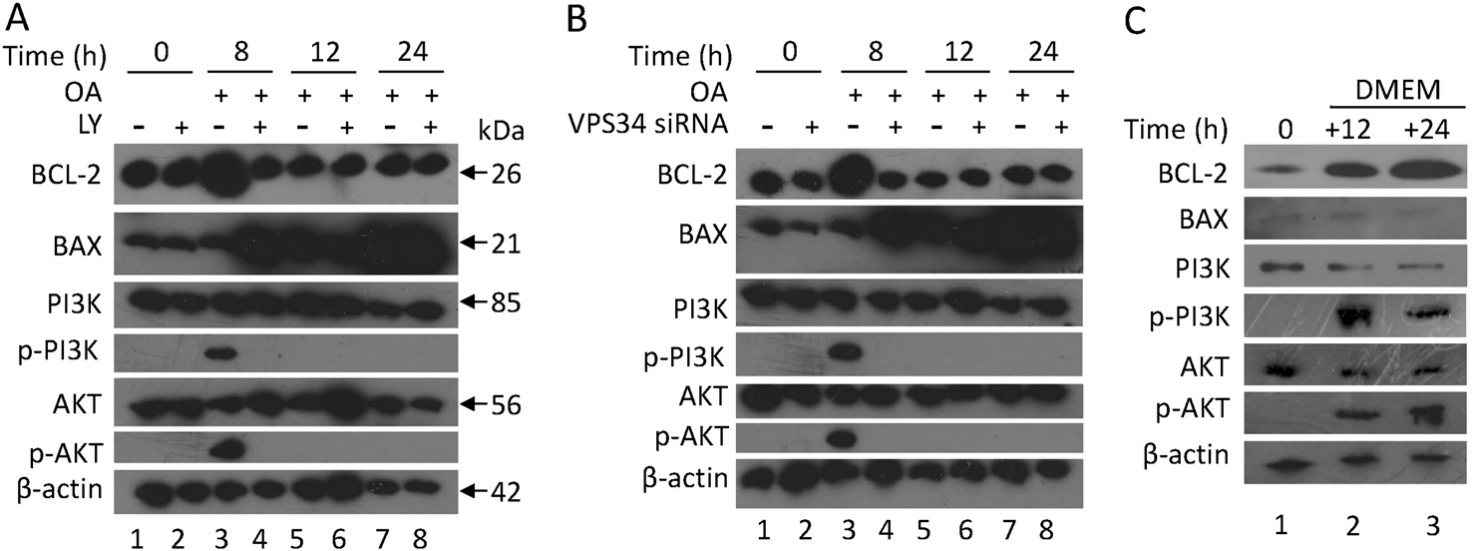
OA treatment activates PI3K/AKT signaling pathway and increases BCL-2 expression at 8 h, whereas increases BAX expression at 24 h HepG2 cells were pre-treated with LY294002 (**a**) or VPS34 siRNA (**b**) and then were cultured with 400 μM OA. **c** HepG2 cells were treated with 400 μM OA for 8 h and then were cultured in normal DMEM culture for 12 and 24 h. **a**–**c** Lysates of HepG2 cells were collected, and an immunoblot assay was conducted with the indicated antibodies

PI3K/AKT signaling pathway activation is associated with an anti-apoptotic function, and the inhibition of the PI3K/AKT pathway contributes to NAFLD development^37^. We sought to determine whether OA treatment could inhibit PI3K/AKT activation and induce apoptosis. Our results showed that phospho-PI3K and phospho-AKT could be detected at 8 h (Fig. 5a, column 3), BCL-2 expression increased at 8 h post-OA treatment (Fig. 5a, column 3), and BAX expression increased at 12 and 24 h (Fig. 5a, columns 5 and 7). Moreover, LY294002 inhibited the activation of the PI3K/AKT pathway, which is shown by the absence of phospho-PI3K and phospho-AKT 8 h post-OA treatment (Fig. 5a, column 4). Using VPS34 siRNA to inhibit autophagy, we obtained similar results to the LY294002-treated samples (Fig. 5b). Thus, our results suggest that the first wave of autophagy induces PI3K/AKT pathway activation. Furthermore, by removing OA after 8 h of treatment, our results show that BCL-2, phospho-PI3K, and phospho-AKT could still be detected 12 and 24 h after the initial exposure to OA (Fig. 5c). These results suggest that the PI3K/AKT pathway remains activated simultaneously with the first wave of autophagy.

### p53-dependent DRAM expression is involved in the second wave of autophagy and contributes to HepG2 apoptosis

**Fig. 6:**
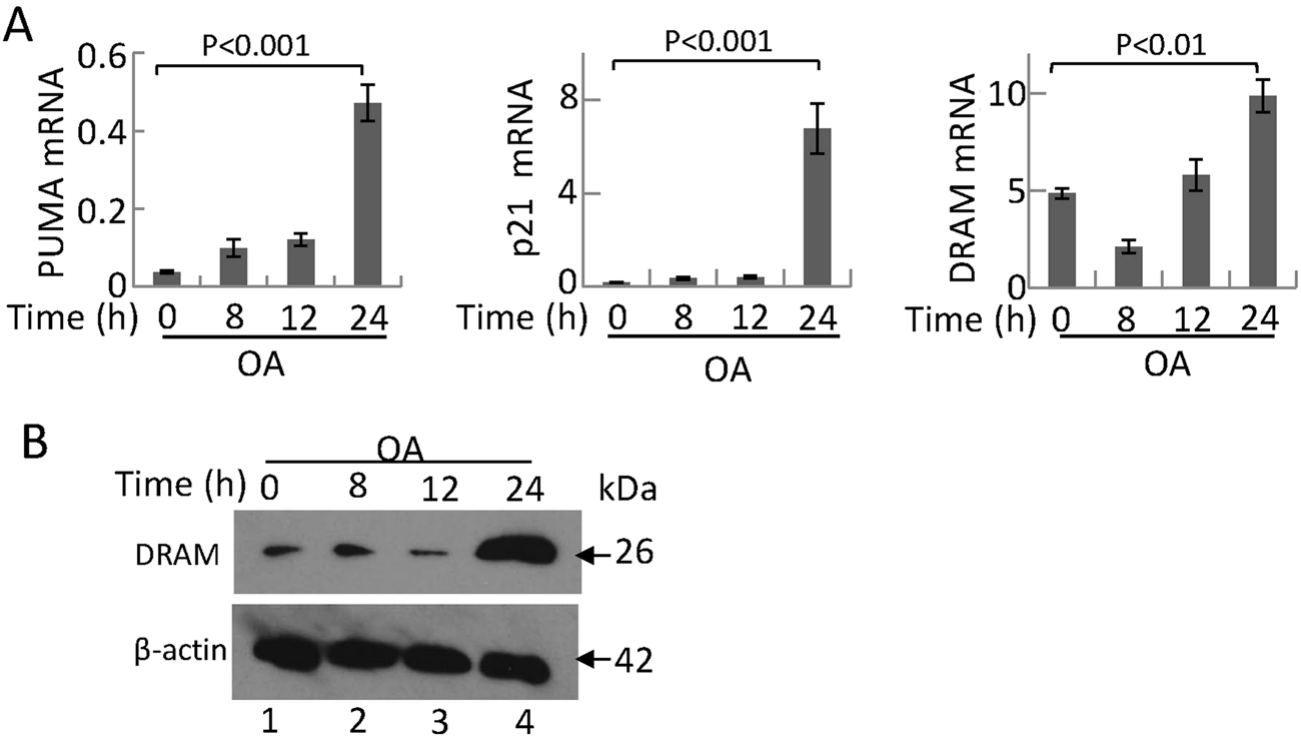
p53 signaling pathway was activated by 400 μM OA treatment **a** Total RNA was isolated from HepG2 cells treated with 400 μM OA. Real-time PCR was employed to detect the mRNA level of PUMA, p21, and DRAM. **b** Lysates of HepG2 cells were collected, and an immunoblot assay was conducted with the anti-DRAM antibody

Previous studies have shown that an imbalance of BCL-2 and BAX expression is involved in hepatocytic apoptosis during NAFLD. BAX is a transcriptional target of p53^38^, and p53 can also regulate the expression of lipid metabolism-associated genes, the dysfunction of which can lead to NAFLD development^39^. The effect of p53 on autophagy is currently unclear: basal levels of p53 function directly in the cytoplasm to inhibit autophagy^21^, whereas some transcriptional targets of p53 (e.g., DRAM) promote autophagy, a response that is consistent with the tumor-suppressive role of p53^40^. Thus, we tried to determine how the activation of p53 signaling contributes to the cellular decision to initiate autophagy or apoptosis. We found that mRNA levels of PUMA, p21, and DRAM increased 24 h post-OA treatment (Fig. 6a). DRAM contributes to cell death by inducing autophagy^41^. Our results showed that DRAM expression was significantly increased after 24 h OA treatment (Fig. 6).

**Fig. 7:**
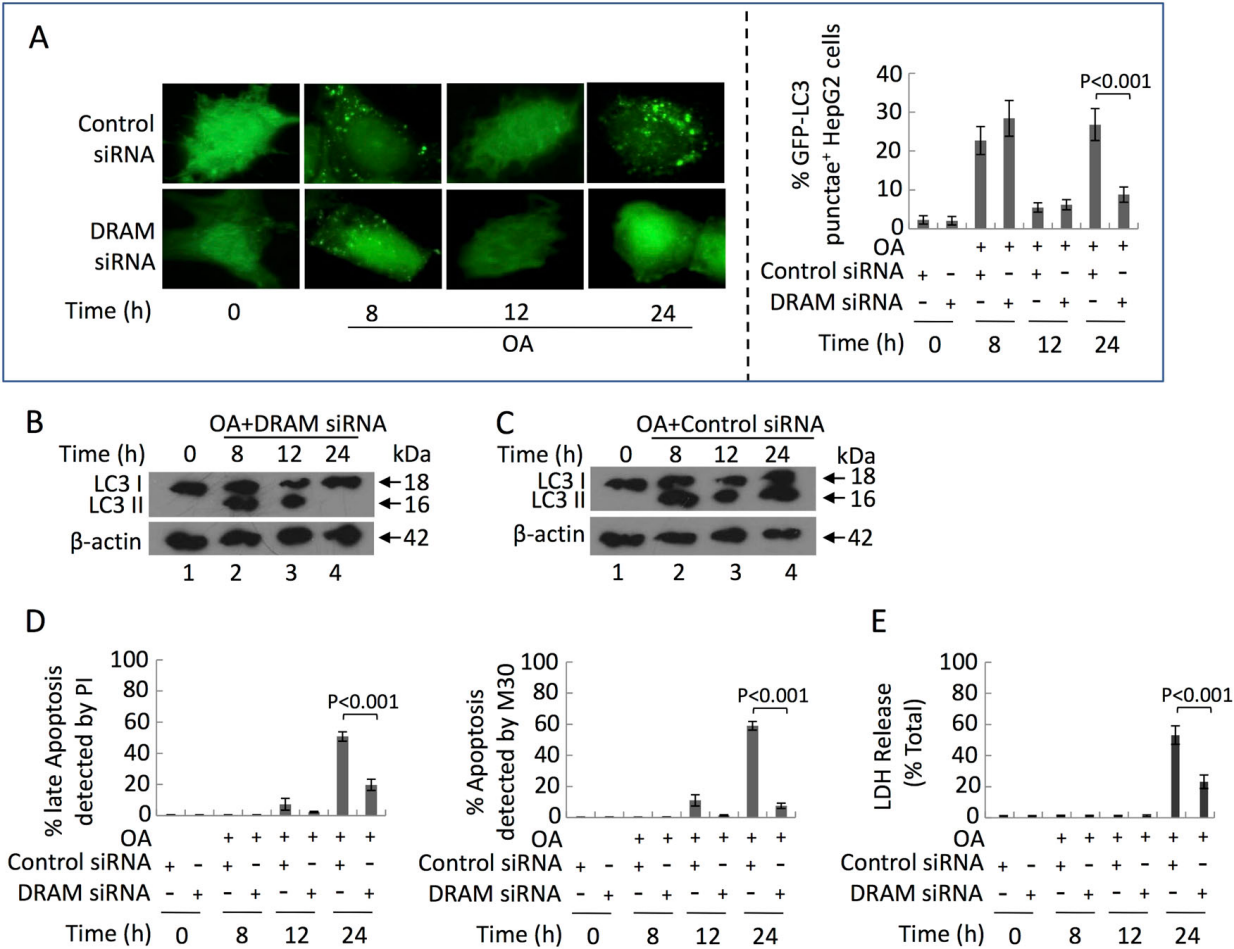
Following 400 μM OA treatment, DRAM expression induced the second wave of autophagy HepG2 cells were pre-treated with DRAM siRNA and then cultured with 400 μM OA. **a** Representative GFP-LC3 images (left panel, original magnification, ×1000), quantification of autophagosome formation (right panel). **b**, **c** Lysates of HepG2 cells were collected, and an immunoblot assay was conducted with the indicated antibodies. **d** Quantification of apoptotic cells by calcein AM/PI and M30 immunoreactivity. **e** The levels of LDH release. **a**, **d**, **e** Data are presented as mean ± SEM in three independent experiments

To further elucidate the role of DRAM, we analyzed the effect of inhibiting DRAM on the progression of autophagy, and we found that DRAM siRNA significantly inhibited autophagy at 24 h but not at 8 h post-OA treatment (Fig. 7a–c). Considering that the second wave of autophagy was mainly composed of mitophagy (Fig. 2e), these results suggest that DRAM plays a key role in inducing mitophagy in HepG2 cells exposed to OA. In fact, our previous data showed that 400 μM OA induced DRAM expression and promoted its mitochondrial localization after 24 h^42^. Interestingly, the PI positive and M30 immunoreactive cells were significantly reduced in DRAM siRNA-treated HepG2 cells at 24 h post-OA treatment (Fig. 7d), and the leakage of LDH decreased dramaticlly at 24 h (Fig. 7e). These results suggest that DRAM-dependent mitophagy cooperates with other p53-dependent apoptotic signals (e.g., PUMA, p21, BAX) to induce apoptosis.

### Autophagy-dependent activation of the PI3K/AKT pathway is present in the liver tissue of patients with low-grade steatosis, and DRAM overexpression was observed in liver tissue of patients with high-grade steatosis

**Fig. 8:**
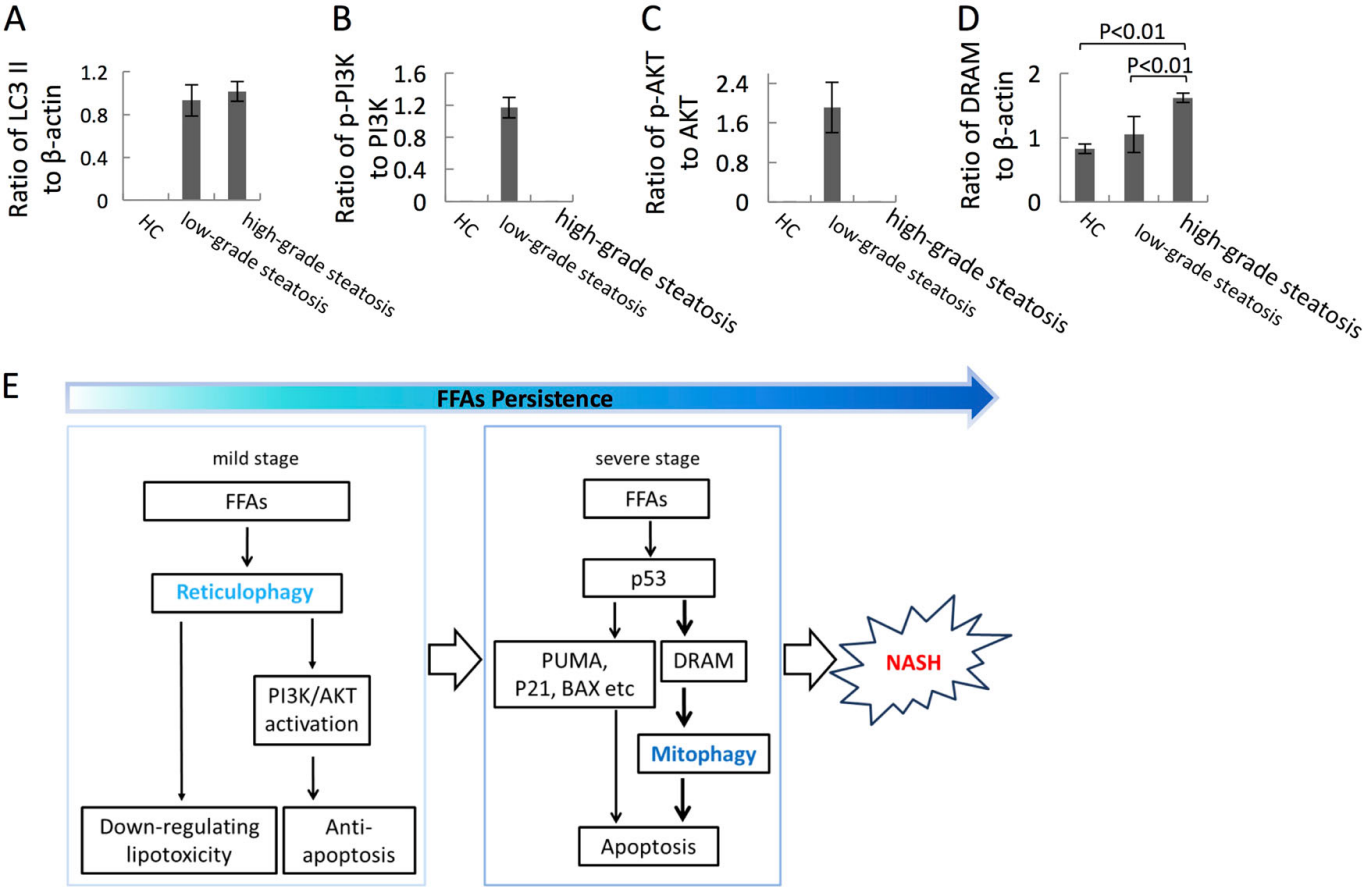
Autophagy development, activation of PI3K/AKT, and DRAM signaling pathways were detected in liver tissues of patients with low-grade steatosis and high-grade steatosis Lysates of liver tissues were collected and immunoblot assay was conducted with the indicated antibodies. Densitometry analysis of the band density ratio of LC3 II (**a**)/DRAM (**d**) to β-actin and phosphor-PI3K (**b**)/-AKT (**c**) to their non-active forms. **e** Model. In the mild stage of NAFLD, FFAs primarily induce reticulophagy to protect hepatocytes via downregulating lipotoxicity and activating the PI3K/AKT signaling pathway. In the severe stage when FFAs are persistent, FFAs induce DRAM expression through p53 and causes hepatocytic apoptosis, through mitophagy in concert with one or more p53-dependent apoptotic signals, leading to NASH development

We observed an induction of autophagy in low-grade steatosis and high-grade steatosis patients (Fig. 8a). Upregulation of M30 has been shown to be associated with NALFD deterioration^28–30^, and our data further confirm this, as we observed increased expression of M30 and elevated mRNA levels of PUMA and p21 in the livers of high-grade steatosis group, suggesting that apoptosis is mainly induced in patients with high-grade steatosis (Supplementary Figure 6). Western blotting results showed that PI3K/AKT phosphorylation was present only in liver samples from patients with low-grade steatosis, and DRAM protein expression was higher in liver samples from patients with high-grade steatosis (Figs 7 and 8). These clinical results support our in vitro findings that autophagy is an independent sequence of events involved in the pathological progression of NAFLD, and autophagy plays different roles as an anti- and pro-apoptotic pathway.

## Discussion

Previous reports have shown that autophagy is impaired in patients with NAFLD. Some factors, such as ER stress, insulin resistance, and mitochondrial dysfunction, have been shown to contribute to the deterioration of NAFLD, and associated with the impairment of autophagy in the context of NAFLD^13^. Thus, the impairment of autophagy appears to be associated with lipotoxicity and the deterioration of NAFLD. OA is a monounsaturated fatty acid, which presents in the diet and in blood plasma. It has been reported that OA had various effects, including stem cell modulation, apoptosis and autophagy, etc.^43,44^. The use of OA building NAFLD model has been successfully demonstrated in some studies; our results showed that reticulophagy and mitophagy were induced in HepG2 upon 8 and 24 h of 400 μM OA treatment, respectively.

Autophagy has been regarded as a type of cell death distinguishable from apoptosis as well as a protective pathway that promotes cell survival^45,46^. Many past studies have focused on a single role of autophagy (initiation or prevention of cell death). Our study is unique in that we help to elucidate the anti- and pre-apoptotic functions of autophagy, which appear to be manifested through reticulophagy and mitophagy. The crosstalk always exists between autophagy and apoptosis, and components of the autophagic pathway may also directly function in the regulation of apoptosis^47,48^. In our study, reticulophagy is identified as a main factor involved in the inhibition of cell death by apoptosis, whereas mitophagy effects are contrary. We think this is a very interesting phenomenon, and may exist in other life processes. In many studies on NAFLD, lipotoxicity-induced ER stress exacerbates NAFLD and leads to the impairment of autophagy^10,49^. Mitophagy is a key factor for reducing the overproduction of reactive oxygen species (ROS), which is a potential inducer of apoptosis^32,50^.

However, our results elucidate previously unknown roles for reticulophagy and mitophagy in the progression of NAFLD. Lipotoxicity-induced reticulophagy appears to primarily prevent hepatocytes from apoptosis; however, continued FFA stimulus appears to prevent reticulophagy from rescuing hepatocytes from lipotoxicity, which leads to an increase in mitophagy. Subsequently, mitophagy promotes the development of hepatocytic apoptosis. It is possible to imply that reticulophagy and mitophagy play a protective or disadvantageous role, respectively, in the NAFLD progression. Previous studies reported that pro- and anti-apoptotic members of the BCL-2 family exert their activities not only at mitochondria but also at the ER^51,52^. According to some studies, mitochondria that undergo permeability transition (PT) with a loss of ΔΨ_m_ are preferentially targeted by mitophagy^53^. Thus, an attractive explanation for our observation is that OA-induced reticulophagy promotes PT-mediated mitophagy development via releasing calcium. However, the exact mechanism by which reticulophagy can be transformed into mitophagy is still unknown.

With respect to the “multiple-hit” hypothesis, lipid accumulation in hepatocytes is sufficient to induce detrimental factors that impair liver function^54^. The levels of lipid droplets and intracellular triglycerides at 8 h of OA treatment significantly increase relative to the basal levels but are significantly less at 24 h, and our results did not show that lipophagy promotes the downregulation of lipotoxicity induced by OA treatment. However, our results showed that reticulophagy is associated with the disappearance of lipid droplets and intracellular triglycerides after OA treatment. An attractive explanation for our observation is that reticulophagy regulates ER homeostasis during the unfolded protein response (UPR), and also reduces triglyceride formation by degrading dysfunctional ER. These results suggest that the different types of autophagy may not involve in the same process and function. Moreover, the findings that the liver tissue of patients with low-grade steatosis accumulates less lipid suggest that autophagy may regulate lipid metabolism during the mNAFLD stage. However, the subtype of autophagy that regulates lipid metabolism in liver tissues of NAFLD patients remains unknown.

In addition to decreasing lipotoxicity, reticulophagy has been associated with the activation of the PI3K/AKT pathway. Some studies have demonstrated that during the development of NAFLD, autophagy is inhibited by the PI3K/AKT signaling pathway via both short-term and long-term regulation mechanisms^13^. However, our results reveal a new relationship between PI3K/AKT signaling pathway activity, reticulophagy, and NAFLD development. Our results suggest that the persistence of reticulophagy in early ER stress is dependent on PI3K/AKT pathway activation. AKT is phosphorylated by PI3K and becomes activated, and phospho-AKT can bind and regulate many downstream effectors such as BCL-2 family proteins^55^. PI3K/AKT activation may promote BCL-2 expression in reticulophagy and may play a protective role during the early stages of NAFLD development.

p53, a recognized tumor suppressor protein, acts as a master regulator with pleiotropic effects on metabolism, anti-oxidant defense, proliferation, senescence, and cell death^56^. Previous data have shown that p53 activation is involved in cell apoptosis during NAFLD^57^, and regulates the balance between BCL-2 and BAX^58^. In this study, our results demonstrate that p53 signaling is active during later stages of OA treatment. The p53 target genes were upregulated in HepG2 cells cultured with OA for 24 h. p21 mediates p53-dependent cell cycle arrest, and PUMA induces apoptosis via interactions with BCL-2 family members^59^.

DRAM is critical for apoptosis, but it has rarely been reported in NAFLD. In this study, the results show that DRAM-mediated autophagy promotes apoptosis, and DRAM expression is only upregulated in the liver tissues of patients with high-grade steatosis. Thus, our data establish a link between p53 activation and mitophagy-mediated apoptosis in hepatocytes via DRAM expression. Because DRAM-mediated mitophagy cannot completely account for the cell apoptosis, we postulate that p21 and PUMA also play roles in inducing hepatocytic apoptosis. Thus, DRAM-mediated autophagy appears to be a critical but not sufficient inducer of apoptosis when p53 is activated in NAFLD.

Taken together, our data suggest that reticulophagy and mitophagy are two independent events during NAFLD pathogenesis (Fig. 8e). Considering the distinct mechanisms and functions displayed by the two waves of autophagy, the different functions of various types of autophagy may offer new insights into the prevention and cure of NAFLD.

## Materials and methods

### Cell culture and treatment

The human hepatoblastoma cell line HepG2 was grown in Dulbecco’s Modified Eagle’s Medium (DMEM) (Invitrogen, Carlsbad, CA) supplemented with 10% fetal bovine serum (FBS) (Invitrogen, Carlsbad, CA). Oleic acid (Sigma-Aldrich, St. Louis, MO) was conjugated to albumin, and cells were treated with 400 μM oleic acid for 24 h. BafA1 (50 nM, Sigma Inc., St. Louis, MO, USA) was used to inhibit the autophagy flux. PI3K inhibitor LY294002 (50 μM) (Cell Signaling Technology, Beverly, MA) was used to inhibit autophagy by pre-treating cells for 5 h. HepG2 cells were transfected with Fugene HD (Promega, Madison, WI) for the transfection of plasmids encoding GFP-LC3 or with Lipofectamine 2000 (Invitrogen, Carlsbad, CA) for the transfection of DRAM siRNA and control siRNA (the sequences of the siRNA were obtained from ref.^38^), VPS34 siRNA (ACGGTGATGAATCATCTCCAA), ATG5 siRNA (GAAGUUUGUCCUUCUGCU), or PINK1 siRNA (CCTCGTTATGAAGAACTAT) (the sequences of the siRNA were obtained from refs.^60,61^. Cells grown on glass cover slips were used for immunofluorescence detection.

### Real-time PCR

The RNeasy Mini Kit (Qiagen, Hilden, Germany) was used to isolate total RNA from cultured cells with or without treatment with OA and/or LY294002. Reverse transcription was conducted using the SuperScript II First-strand Synthesis System for RT-PCR (Invitrogen, Carlsbad, CA) to synthesize first-strand cDNA. SYBR Green was used to detect dsDNA product during the real-time PCR reaction. The mRNA content was normalized to the housekeeping gene β-actin. Specific primer sequences used for real-time PCR were as follows: for PUMA, 5′-CGACCTCAACGCACAGTACGAG-3′ (forward) and 5′-AGGAGTCCCATGATGAGATTGTACA-3′ (reverse); for p21, 5′-CAGGCTGAAGGGTCCCCAGGTGGA-3′ (forward) and 5′-GGATTAGGGCTTCCTCTTGGAGA-3′ (reverse); for DRAM, 5′-TCAAATATCACCATTGATTTCTGT-3′ (forward) and 5′-GCCACATACGGATGGTCATCTCTG-3′ (reverse) (the sequences of DRAM primers were obtained from ref.^38^); for β-actin, 5′-GCCCTGAGGCACTCTTCCA-3′ (forward) and 5′-CGGATGTCCACGTCACACTT-3′ (reverse).

### Subcellular fractionation and western blotting

Subcellular fractionation of HepG2 cells was conducted as described previously^51^. Briefly, the lysates of HepG2 cells were centrifuged at 1000 × *g* for 30 min and the pellet was considered nuclei-enriched fragment. The nuclei-depleted supernatant was centrifuged at 10,000 × *g* for 30 min and the pellet was considered mitochondria-enriched fragment. The mitochondria-depleted supernatant was further centrifuged at 100,000 × *g* for 2 h to yield the ER-enriched pellet. Lysates of cells and liver tissues were subjected to western blot analysis, as previously described^34^. Briefly, total cellular lysates were separated by 10% or 12% SDS-PAGE and then were transferred to PVDF membranes. The membranes were blocked with 5% milk, and were probed sequentially with specific primary antibodies and horseradish peroxidase-conjugated secondary antibodies (all antibodies were purchased from Santa Cruz Biotechnology (Santa Cruz, CA, USA) except anti-actin antibody (Sigma-Aldrich, St. Louis, MO)).

### Fluorescence microscopy and apoptosis assay

Frozen cells were fixed with 10% paraformaldehyde/PBS, incubated in 1% Triton X-100/PBS for 5 min, blocked with 3% BSA/PBS, and probed with mouse anti-M30 antibody which was produced by our laboratory and is used for detecting early apoptosis^62^. Cy3-conjugated anti-mouse secondary antibodies (Sigma-Aldrich, St. Louis) were used to amplify the signal. Nuclei were counterstained with 4,6-diamidino-2-phenylindole (DAPI) (Thermo Fisher, Eugene, Oregon, USA). Some cells were rinsed with 1 × PBS once and then were incubated for 15 min with propidium iodide (PI) and calcein acetoxymethyl ester (calcein-AM) (Invitrogen, Carlsbad, CA) to detect cells in the late stage of apoptosis. M30 immunoreactivity and PI staining were detected using a fluorescence microscope (Nikon Eclipse 80i). Quantitative apoptosis analysis was performed by counting more than 1000 cells in each sample.

### Live-cell imaging

Detection and colocalization of mitochondria with lysosomes or ER with lysosomes was done. HepG2 cells were cultured on coverslips inside a Petri 3.5 cm dish. When cells have reached the desired confluence, remove the medium from the dish and add the pre-warmed (37 °C) probe-containing medium. Incubate (37 °C, 5% CO_2_) the cells for 30 min, then replace the loading solution with a fresh medium and observe the cells using a confocal microscope (Leica TCS SP8, Germany) fitted with the correct filter set. ER-Tracker Green (BODIPY FL glibenchamide), LysoTracker Red DND-99, and MitoTracker Deep Red FM were purchased from Thermo Fisher (Eugene, Oregon, USA). We used working concentrations of 1 μM, 75 nM, and 400 μM for ER-Tracker, LysoTracker, and MitoTracker, respectively.

### LDH assay

The level of LDH in the supernatant of cultured cells was detected using an LDH assay kit (Applygen Technologies Inc., Beijing, China) according to the manufacturer’s recommended protocol^63^.

### Intracellular triglyceride assay

The concentration of intracellular triglycerides was estimated using an ultrasensitive assay kit for FFAs (Applygen Technologies Inc., Beijing, China) according to the manufacturer’s recommended protocol^63^.

### Oil Red O staining

HepG2 cells were washed twice with 1 × PBS and were fixed with 4% paraformaldehyde for 15 min. After three washes with PBS, cells were stained for 30 min in a freshly diluted Oil Red O solution. The dishes were rinsed in 1 × PBS and then representative images of lipid droplets stained with Oil Red O were obtained by microscopic imaging.

### Patients and immunohistochemistry

Patients with low-grade steatosis (*n* = 6) and high-grade steatosis (*n* = 6) NAFLD were recruited from You'an Hospital in Beijing. Normal liver tissues were obtained from resected liver metastases, which were used as healthy controls (HC, *n* = 4). The patients were diagnosed NAFL and excluded NASH via pathological and serological diagnosis. Our hospital doctors distinguished low-grade steatosis from high-grade steatosis by type-B ultrasonic and HE staining of the pathological section; steatosis less than 30% was considered as low-grade steatosis and greater than 60% as high-grade steatosis. Liver tissues were fixed in 10% paraformaldehyde and then were embedded in paraffin. A 4-μm-thick section cut from a paraffin-embedded block was stained with mouse monoclonal antibodies specific for M30. Briefly, liver sections were deparaffinized in xylene and were rehydrated in graded ethanol. The tissues were pre-incubated with 3% horse serum for 30 min to prevent non-specific antibody binding. Primary antibodies were diluted at 1:250 and were used to stain the tissues. The proteins were extracted from the paraffin-embedded liver tissues. Our protocol was approved by the ethics committee of You'an Hospital in Beijing, and all patients provided the informed consent for participation in this study.

### Statistical analysis

All data represent at least three independent experiments and are expressed as the mean ± SEM. Differences between the groups were compared using the Student’s *t*-test or ANOVA. Differences were considered significant if the *p*-value was less than 0.05.

## Electronic supplementary material

Supplementary figures and legends
