# Supplementary material for: Differential effects of reticulophagy and mitophagy on nonalcoholic fatty liver disease
Source: Cell Death Dis. 2018 Jan 24;9(2):90. doi: 10.1038/s41419-017-0136-y (PMC5833629; doi:10.1038/s41419-017-0136-y)
Supplement: Supplementary file 1 — Supplementary figures and legends [file 41419_2017_136_MOESM1_ESM.docx]

**Supplementary figures and legends**


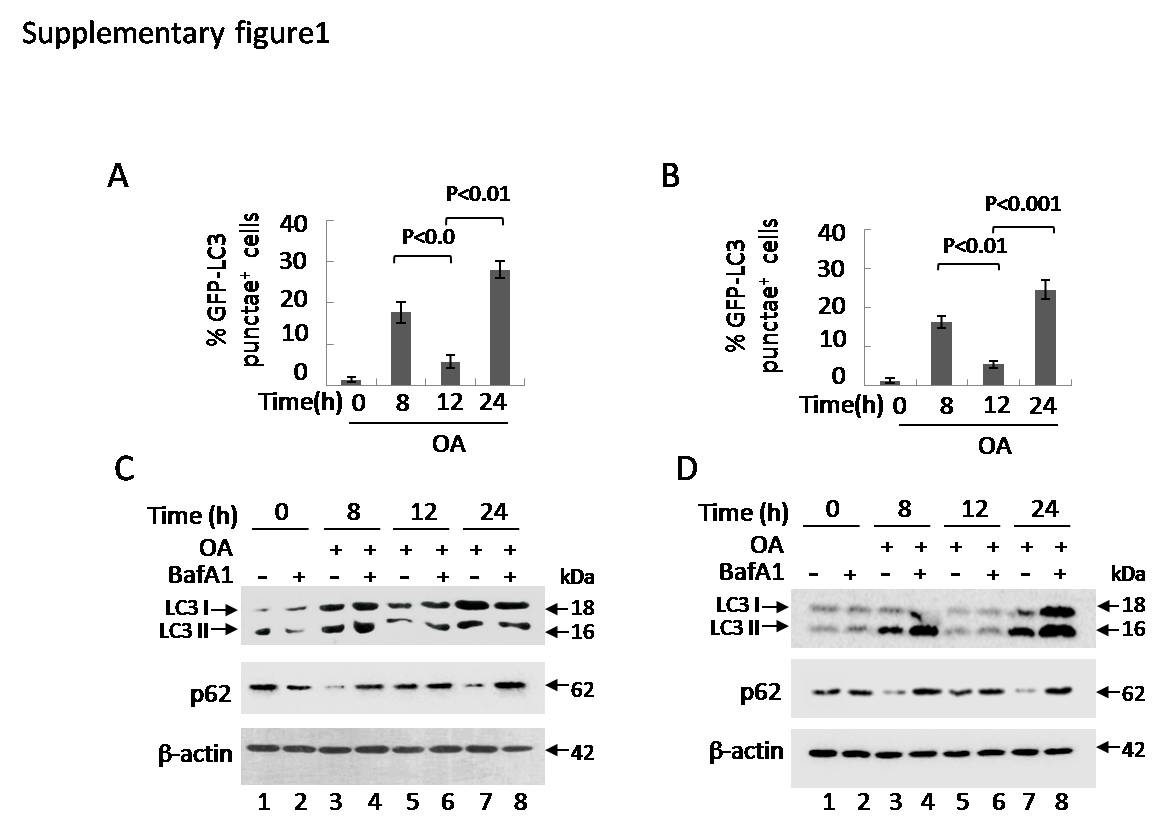


Supplementary figure 1. Autophagy development peaks at 8 and 24 hours in response to 400μM OA stimulus in 7702 and SMMC7721 cells. (A and B) The percentage of autophagosome of 7702(A) and SMMC-7721 cells(B), cells with 5 or more GFP-LC3 puncta were considered to have accumulated autophagosomes. Data are presented as mean ± SEM in three independent experiments. (C and D) Representative western blotting analysis of LC3I/II and p62 expressions after using Bafilomycin A1 in 7702(C) and SMMC-7721 cells(D).


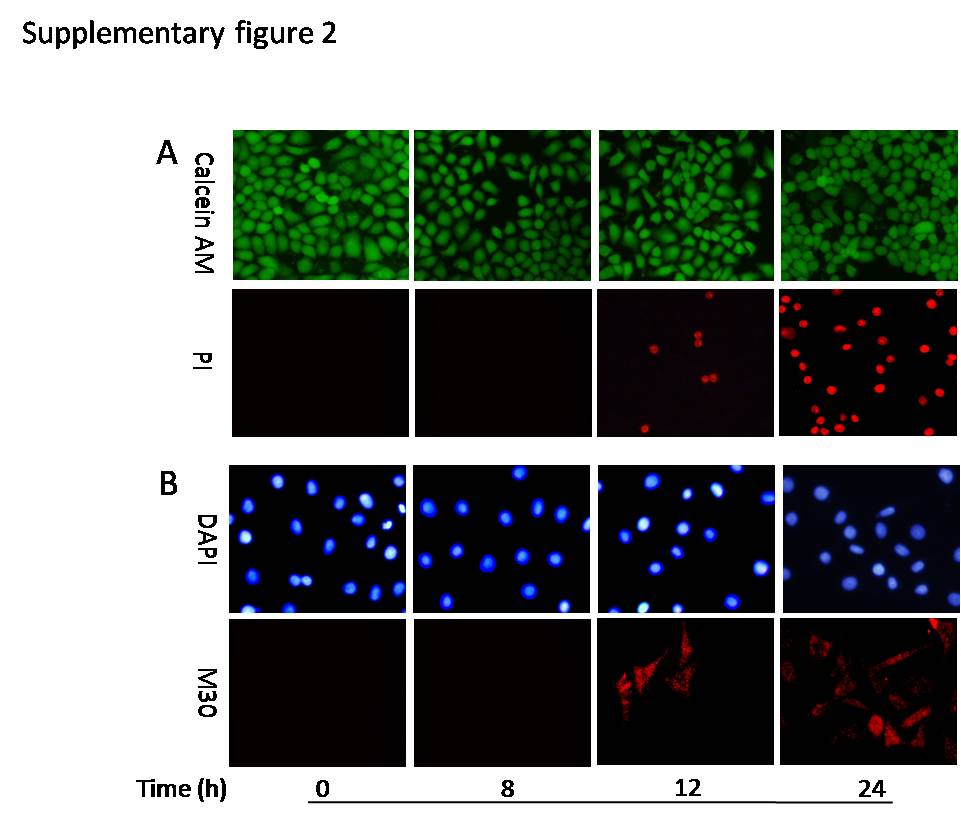


Supplementary figure 2. (A and B) Representative images of Calcein AM/PI staining (A, upper panel) and DAPI/M30 staining (B, lower panel), Original magnification, ×400.

**
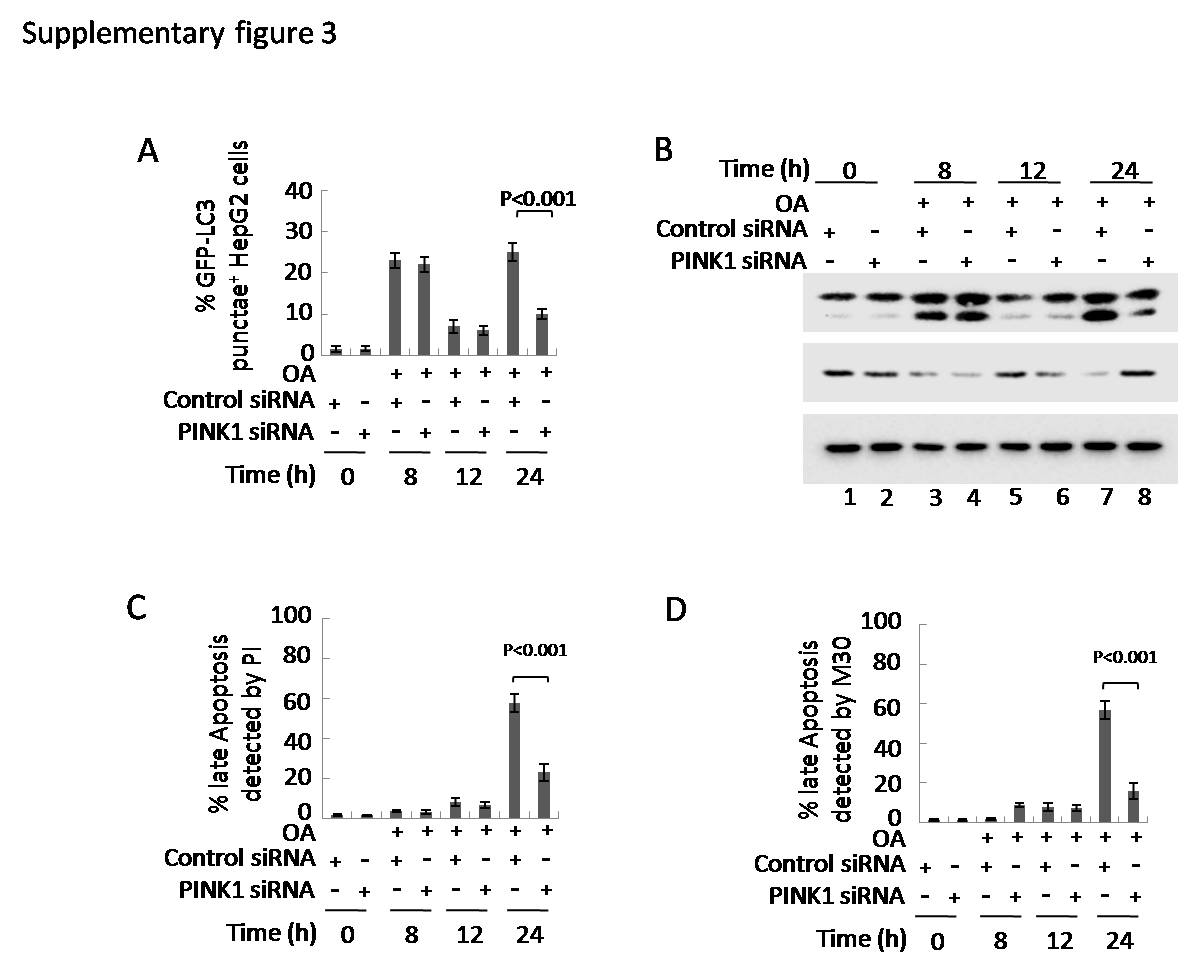
**

Supplementary figure 3. Following 400μM OA treatment, PINK1 knockdown inhibited the second wave of autophagy. (A) Quantification of autophagosome formation, cells with 5 or more GFP-LC3 puncta were considered to have accumulated autophagosomes. (B) Representative western blotting analysis of LC3I/II and p62 expressions after using Bafilomycin A1 in HepG2 cells. (B and C) Quantification of apoptotic cells by Calcein AM/PI(B) and M30(C) immunoreactivity. (A, B and C) Data are presented as mean ± SEM in three independent experiments.


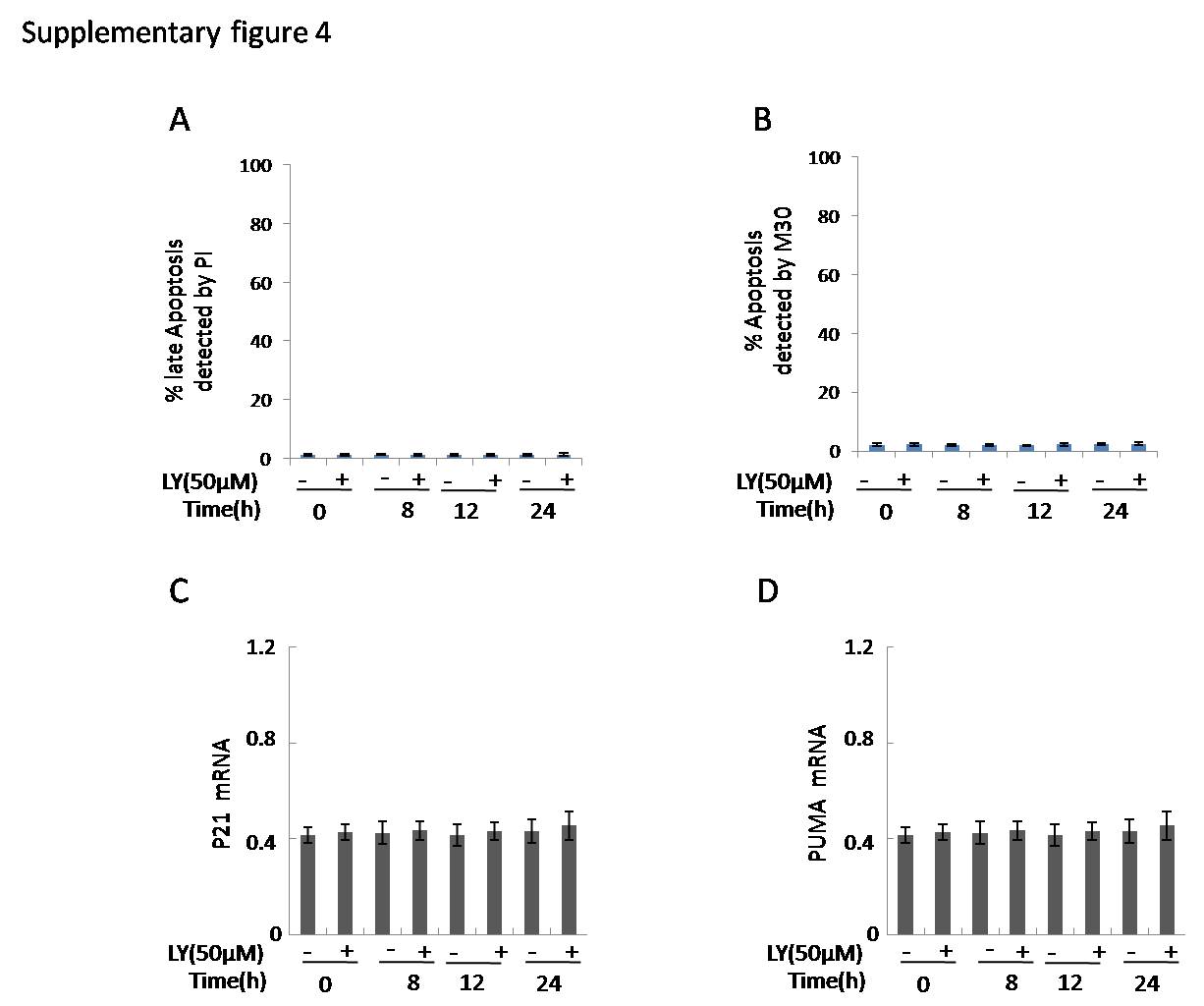


Supplementary figure 4. LY294002 (LY) pretreatment can not induce apoptosis solely. (A and B) HepG2 cells were pretreated by LY294002 (50μM) for 5 hours and then were grown in culture medium with or without 400μM OA. Quantification of apoptosis by PI staining (A) and M30 immunoreactivity (B) as mean % apoptotic cells ± SEM in three independent experiments. (C and D ) Real time PCR analysis of mRNA levels of p21 and PUMA in cells. Data are presented as mean ± SEM in three independent experiments.


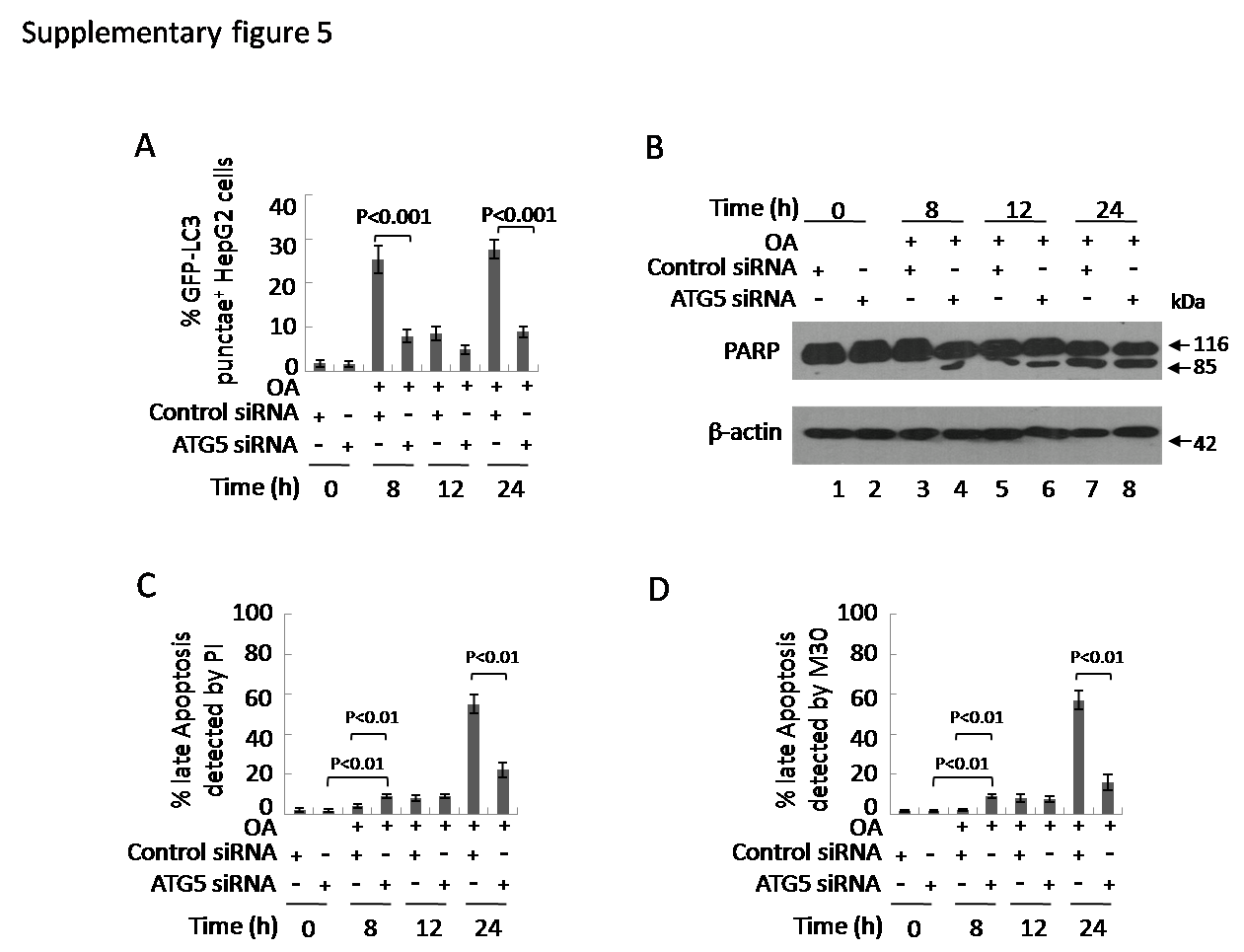


Supplementary figure5. ATG5 siRNA significantly inhibited autophagy in HepG2 cells at 8 and 24 hours post-OA treatment, and inhibited cell apoptosis distinctly at 24 hours. We transfected HepG2 cells with GFP-LC3-expressing plasmid and ATG5 siRNA. The count of GFP-LC3 punctae+ cells (A), quantification of late (B) and early (C) apoptosis of cells by PI staining and M30 immunoreactivity, respectively. Data are presented as mean ± SEM in three independent experiments.


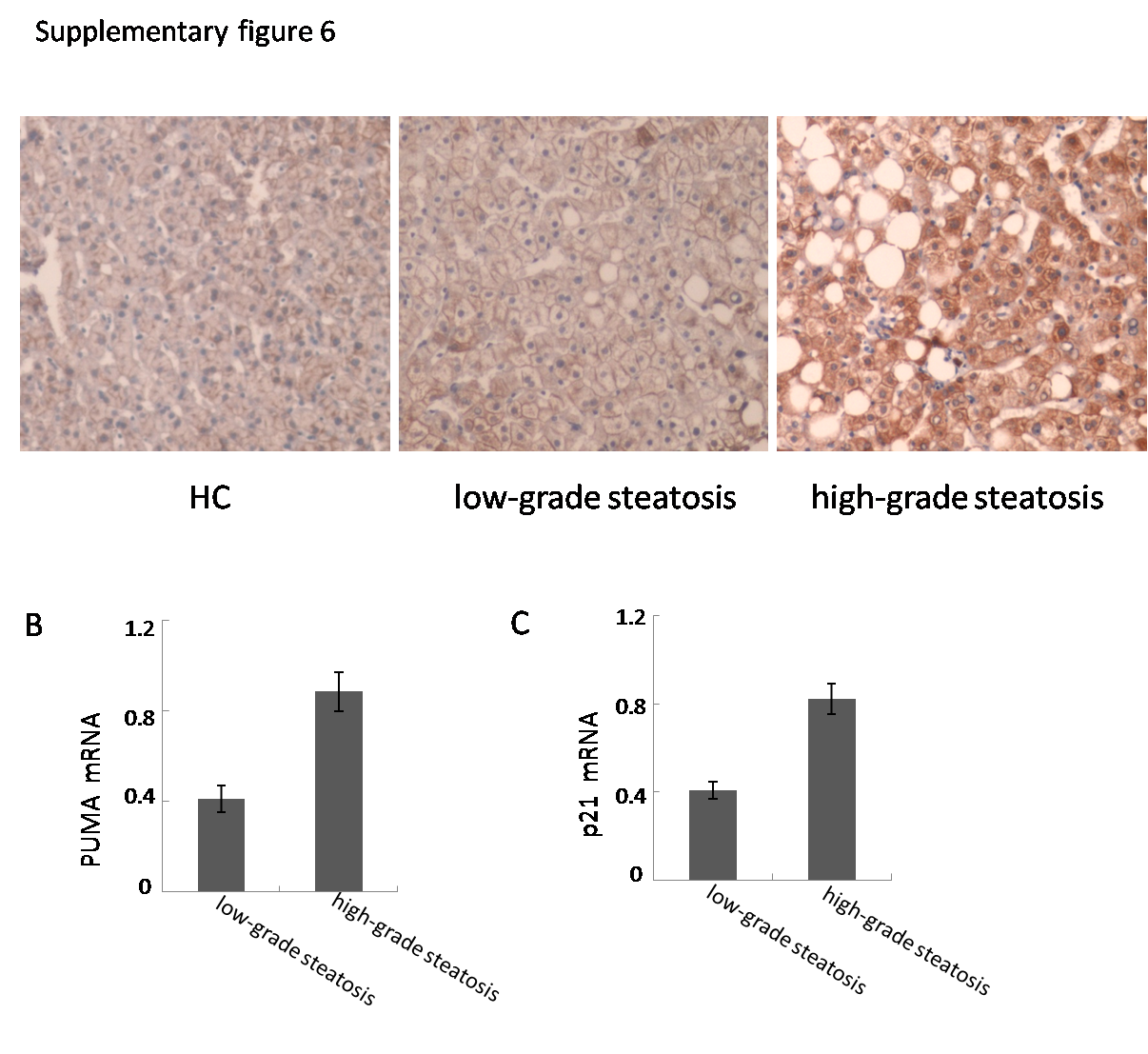


Supplementary figure 6. Liver tissues of high-grade steatosis patients showed high M30 immunoreactivity. (A) Liver tissues from low-grade steatosis (n=6) and high-grade steatosis (n=6) patients were used to detect M30 immunoreactivity. Normal liver tissues obtained from resection of liver metastases were used as healthy control (HC, n=4). Original magnification, ×100. (B) Real time PCR analysis of mRNA levels of PUMA and p21.Data are presented as mean ± SEM in three independent experiments.


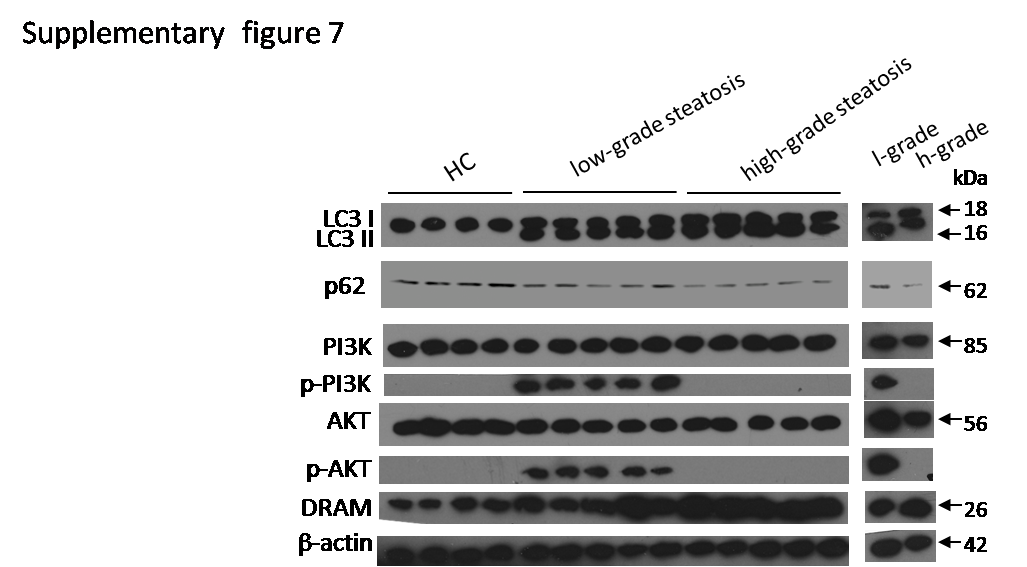


Supplementary figure7.Autophagy development, activation of PI3K/AKT and DRAM signaling pathways were detected in livers tissues of patients with low-grade steatosis (n=6) and high-grade steatosis (n=6) by western blotting assay. Normal liver tissues obtained from resection of liver metastases were used as healthy control (HC, n=4).
